# Incidence of hypertension in a prospective cohort study of adults from Porto, Portugal

**DOI:** 10.1186/1471-2261-12-114

**Published:** 2012-11-28

**Authors:** Marta Pereira, Nuno Lunet, Cristiana Paulo, Milton Severo, Ana Azevedo, Henrique Barros

**Affiliations:** 1 Department of Clinical Epidemiology, Predictive Medicine & Public Health, Institute of Public Health of the University of Porto (ISPUP), University of Porto Medical School, Porto, Portugal; 2 Department of Clinical Epidemiology, Predictive Medicine & Public Health, Institute of Public Health of the University of Porto (ISPUP, University of Porto Medical School, Porto, Portugal; 3 Department of Internal Medicine, Centro Hospitalar São João, Porto, Portugal; 4 Department of Clinical Epidemiology, Predictive Medicine & Public Health, Institute of Public Health of the University of Porto (ISPUP), University of Porto Medical School, Porto, Portugal; 5 Department of Clinical Epidemiology, Predictive Medicine & Public Health, Institute of Public Health of the University of Porto (ISPUP), University of Porto Medical School, Porto, Portugal; 6 Department of Clinical Epidemiology, Predictive Medicine & Public Health, Institute of Public Health of the University of Porto (ISPUP), University of Porto Medical School, Porto, Portugal

**Keywords:** Adults, Hypertension, Incidence, Portugal

## Abstract

**Background:**

During the past 30 years, Portugal has been described as one of the countries with highest median blood pressure levels in Europe, but the incidence of hypertension is unknown. The aim of this study was to estimate the incidence of hypertension, according to socio-demographic characteristics and lifestyles.

**Methods:**

A population-based cohort of randomly selected dwellers from Porto, Portugal, aged ≥18 years, was assembled in 1999–2003 (EPIPorto study) and 796 hypertension-free individuals (62.6% women) were reassessed after a median of 3.8 years. Hypertension was defined as blood pressure ≥140/90 mmHg and/or antihypertensive drug therapy. Incidence rate ratios (IRR) were estimated using Poisson regression.

**Results:**

The overall incidence rate was 47.3 [95% confidence interval (95% CI): 40.5-55.5] per 1000 person-years. Among women, the incidence was 43.4 (35.6-53.1) and among men 52.7 (41.3-68.0) per 1000 person-years. The incidence was lower in women up to 60 years and much higher among women above 60 (110.0 *vs.* 64.4 per 1000 person-years among men, p for age-sex interaction=0.032). Participants with higher education had a lower risk of becoming hypertensive (≥13 years *vs.* ≤4 years: RR=0.70, 95% CI, 0.46-1.08, p for linear trend <0.001), independently of age and sex. Overweight and obesity were associated with a 1.67-fold and 2.44-fold increased risk of hypertension, respectively, independently of age, sex and education.

**Conclusions:**

In this urban Portuguese population the incidence rate of hypertension was high, with new cases occurring predominantly among older subjects, the less educated and those with overweight-obesity. Despite recent progresses in blood pressure related outcomes, the risk of hypertension remains higher in Portugal than in other developed countries.

## Background

Hypertension is a modifiable risk factor responsible for a high burden of disability and death [1,2]. Beyond genetic susceptibility, environmental factors change the severity of blood pressure elevation and the timing of hypertension onset [3,4]. There is a high lifetime risk of hypertension, which is sustained after middle age. In the Framingham Heart Study, the residual lifetime risk of hypertension for middle-aged and elderly individuals was 90%, and more than half of 55-year-old participants and about two thirds of 65-year-old participants developed hypertension within 10 years [5].

In Portugal, where stroke has been the leading cause of death for decades, hypertension is a highly prevalent condition [6,7]. According to two population-based studies in the early 2000s, describing the adult population of mainland Portugal [7] and of Porto [8], the prevalence of hypertension was approximately 40%. The prevalence of self-reported hypertension has been increasing in Portugal for over two decades [9]. However, this can result from higher diagnostic sensitivity, or a higher awareness of providers or patients, since mean blood pressure levels have been decreasing for at least 30 years and the prevalence of hypertension, objectively defined by high blood pressure levels or being under antihypertensive treatment, is stable in younger ages and decreasing in older people [9].

Despite its usefulness as a measure of the burden of disease, the prevalence of hypertension is largely determined by the proportion of subjects under treatment and longer duration of cases [10]. On the other hand, hypertension incidence, although influenced by the access of the population to diagnosis, reflects more closely the scope for primary prevention, since it represents the rate of occurrence of new cases. However, population health data seldom provide such information.

Thus, we aimed to quantify the incidence of hypertension in adults, based on the prospective study of a community cohort of Porto, Portugal inhabitants, and to compare rates according to socio-demographic and behavioural characteristics.

## Methods

A cohort of 2485 adult dwellers in Porto, an urban center in the northwest of Portugal with almost 300,000 inhabitants at that time, was assembled between 1999 and 2003 [8,11]. Briefly, simple random digit dialing of landline telephones was used to select households. The vast majority of houses (>95%) had a landline telephone at the time of this procedure. We used a table of random numbers to define the last four digits that are specific to individual houses, assuming the local prefix codes to limit the universe to the city of Porto. Non-existing numbers, those corresponding to fax numbers or telephone numbers of non-individual subscribers were ignored. The household was considered unreachable after at least four dialing attempts at different hours and including week and weekend days. Within each household, we selected a permanent resident aged 18 years or more using simple random sampling. We considered a refusal if the person explicitly said that she did not want to participate and refusals were not substituted within the same household. The proportion of participation was 70% [12]. A follow-up evaluation was conducted from 2005 to 2008, by trained interviewers, using structured questionnaires and forms, following the same protocol for data collection as at baseline. In both evaluations, participants were invited to visit our Department for an interview, which included a questionnaire on social, demographic, behavioural and clinical data, and a physical examination, including measurement of weight, height and blood pressure.

Age was categorized in 3 groups: <40, 40–60, 60 years or more. Education was recorded as completed years of schooling and subjects divided into three categories: ≤4, 5–12 and ≥13 years. Marital status was self-reported and grouped into two classes: married or in a civil union and single, widowed or divorced/separated.

We considered current smokers those who reported to smoke daily (at least one cigarette per day at the time of the survey) or occasionally (less than a cigarette per day) and ex-smokers those who had stopped smoking for at least 6 months. Average lifetime alcohol consumption, considering duration, frequency, and amount consumed by type of alcoholic beverage (wine, beer, spirits and liquors) was assessed using a questionnaire. A photographic album was used to assist in the report of the average size of the drinks. The daily intake of ethanol (g/day) was estimated by multiplying the quantity and frequency of intake of each drink by its alcohol content. Classes of alcohol consumption were defined by the cut-off points 15.0 g/day for women and 30.0 g/day for men, according to the recommendations of the American Heart Association [13].

Physical activity was evaluated using a previously validated questionnaire [14] exploring all professional, domestic and leisure-time activities, detailing the intensity, duration and frequency of each activity. We used metabolic equivalents (MET), defined as the ratio between the metabolic rate during a specific physical activity and a reference metabolic rate, to quantify the intensity of each activity. The intensity of each activity was then multiplied by its frequency and duration to obtain the amount of physical activity measured as MET*hour/day. The subjects were then classified according to the sex-specific tertiles of the sample distribution.

Anthropometric measurements were performed after an overnight fast, with the participant wearing light clothing and no footwear. Body weight was measured to the nearest 0.1 kg using a digital scale, and height was measured to the nearest centimeter in the standing position using a wall stadiometer. Body mass index (BMI) was calculated as weight (kg) divided by squared height (m^2^) and subjects categorized according to the recommendations of the World Health Organization: obese (≥30 kg/m^2^), overweight (25.0–29.9 kg/m^2^), normal (18.5–24.9 kg/m^2^) and underweight (<18.5 kg/m^2^) [15]. The small number of underweight participants in this study did not allow accurate inferences about this group and therefore this category was grouped with normal weight.

Blood pressure measurements were performed according to the recommendations of the American Heart Association valid at the time of data collection [16]. Participants were instructed to take their usual medication and restrain from alcohol, tea, coffee, smoking or practise exercise in the 30 minutes preceding the measurement. Systolic blood pressure was identified by phase I Korotkoff sound and diastolic blood pressure by phase V. Two measurements of blood pressure separated by at least 5 minutes were taken, on a single occasion, with a mercury sphygmomanometer after 10-minute rest, with no tight clothes, on the right upper arm and at the heart level. The mean was considered and when the difference was larger than 5 mmHg for systolic or diastolic blood pressure a third measurement was taken and the mean of the 2 closest values was registered. The average of the readings was used to classify participants into one of the blood pressure categories of the 2007 Guidelines for the Management of Arterial Hypertension of the European Society of Hypertension and the European Society of Cardiology: optimal (systolic <120 mmHg and diastolic <80 mmHg), normal (systolic 120–129 mmHg or diastolic 80–84 mmHg), high normal blood pressure (systolic 130–139 mmHg or diastolic 85–89 mmHg) [17]. Arterial hypertension was defined as systolic blood pressure ≥140 mmHg and/or diastolic blood pressure ≥90 mmHg and/or current antihypertensive drug therapy [17,18]. Blood pressure was measured following the same technical procedures at baseline and follow-up assessments.

**Figure 1:**
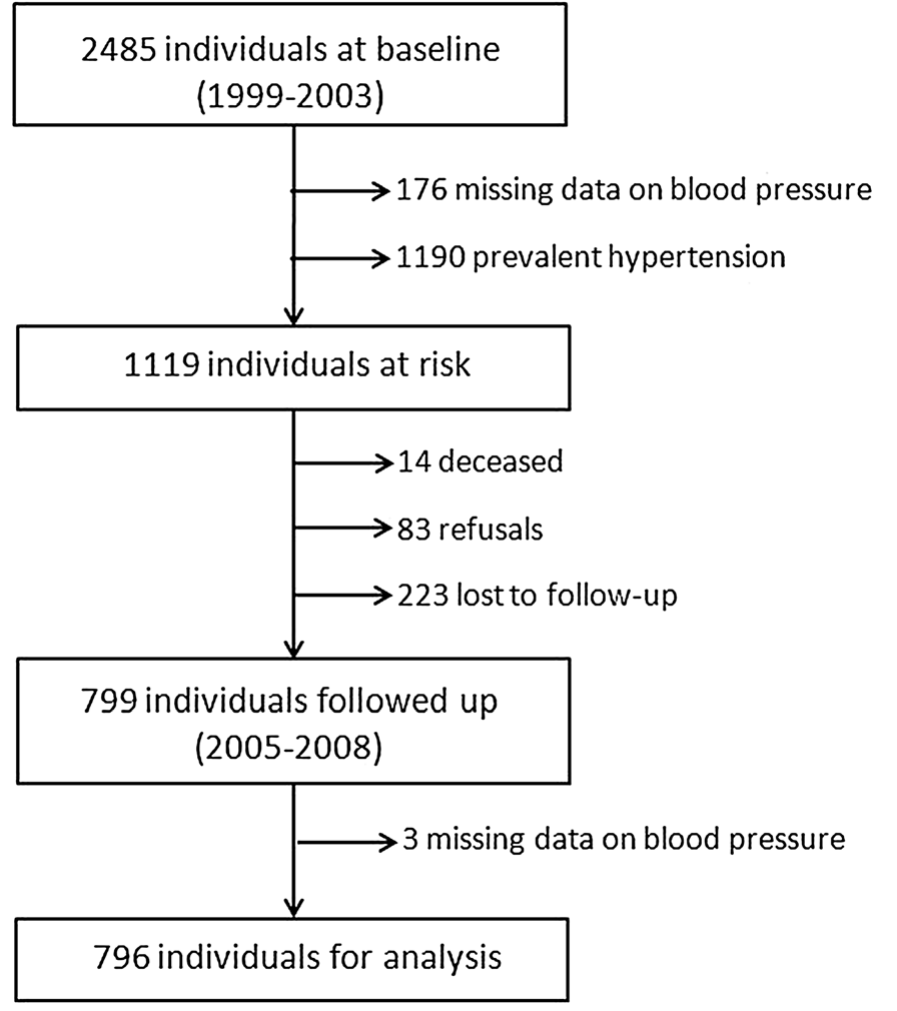
Flowchart illustrating the sample selection for the present analysis.

Among the 2485 individuals evaluated at baseline, 1366 were not eligible for the present study due to missing information on blood pressure (n=176) or prevalent hypertension at baseline (n=1190). From the remaining 1119 individuals considered for the present longitudinal analysis, 320 did not attend the complete follow-up evaluation (Figure 1). Three participants had missing data on blood pressure at follow-up. Participants with available data from the follow-up visit were more likely to be older, to be married or in civil union, less likely to be current smokers and more likely to have high normal blood pressure at baseline (Table 1).

**Table 1:** Comparison of the participants with and without available data, among those eligible for the analysis

	**With available data N=796 n (%)**	**Without available data N=323 n (%)**	**P value**
**Female gender**	498 (62.6)	214 (66.2)	0.245
**Age (years)**			
<40	255 (32.0)	140 (43.3)	0.001
40–60	433 (54.4)	141 (43.6)	
≥60	108 (13.6)	42 (13.0)	
**Education**			
≤4	194 (24.4)	86 (26.6)	0.552
4–12	300 (37.7)	125 (38.7)	
≥13	302 (37.9)	112 (34.7)	
**Married/civil union**	533 (67.0)	195 (60.6)	0.042
**Tobacco consumption**			
current smoker	242 (30.5)	124 (39.0)	0.019
ex-smoker	151 (19.0)	48 (15.1)	
never-smokers	401 (50.5)	146 (45.9)	
**Ethanol intake ≥15.0 g/day (women) or ≥30.0 g/day (men)**	193 (26.1)	67 (23.2)	0.331
**Physical activity**^**1**^			
1^st^ third	262 (33.2)	99 (31.1)	0.674
2^nd^ third	257 (32.6)	112 (35.2)	
3^rd^ third	269 (34.1)	107 (33.6)	
**Body mass index (Kg/m**^**2**^**)**			
<25	411 (52.4)	178 (56.7)	0.284
25–29.9	281 (35.8)	108 (34.4)	
≥30	92 (11.7)	28 (8.9)	
**Blood pressure at baseline**^**2**^			
Optimal	286 (35.9)	121 (37.5)	0.043
Normal	266 (33.4)	126 (39.0)	
High normal	244 (30.6)	76 (23.5)	

^1^ The tertiles of the sample distribution of physical activity were 1.41, 1.58 and 3.46 MET*hour/day among women and 1.38, 1.61 and 3.26 MET*hour/day among men;

^**2**^ Optimal blood pressure was defined as systolic <120 mmHg and diastolic <80 mmHg, normal blood pressure as systolic 120–129 mmHg or diastolic 80–84 mmHg and high normal blood pressure as systolic 130–139 mmHg or diastolic 85–89 mmHg. If systolic and diastolic blood pressure readings belonged to different categories, the highest was considered.

Among the 796 respondents, 62.6% were women, the median schooling was 11 years and one quarter did not complete more than elementary school, 12% were obese, most were sedentary and the median level of total physical activity was 1.47 MET*hour/day. The median ethanol intake was 26.3 g/day among men and 1.4 g/day among women, with 197 (41.7%) women reporting no alcohol intake at all.

### Statistical analysis

The incidence rate was computed as the number of new cases of hypertension over the total person-time at risk. Time at risk was counted as the time between the two evaluations for subjects who remained free of hypertension; in 123 new cases of hypertension (excluding those who started antihypertensive drugs) time at risk was extrapolated by the proportional variation of blood pressure, assuming this variation was linear in time, and for the 62 individuals who were started on antihypertensive drugs the medium time between the two evaluations was assumed as the date of onset. We used this methodology to take into account that those with higher baseline blood pressure have a higher probability of becoming hypertensive for a given time period. The median (interquartile range) of the follow-up period was 3.8 (2.4-6.4) years.

Poisson regression was used to compute relative risks (RR) and respective 95% confidence intervals (95% CI) to quantify the association between explanatory variables and hypertension incidence. The possible interaction of each of the other explanatory variables with age was tested. Crude and sex-, age- and education-adjusted RR were estimated, considering the interaction that was documented between sex and age. All analyses were weighted according to the known age and gender structure of the population of Porto, obtained from census data in 2001, to attain estimates generalizable to this population. The statistical analysis was performed using Stata version 9® (Stata Corporation, College Station, Texas, USA).

### Ethics

The ethics committee “Comissão de Ética para a Saúde” of Hospital de São João approved the study and participants provided written informed consent.

## Results

**Figure 2:**
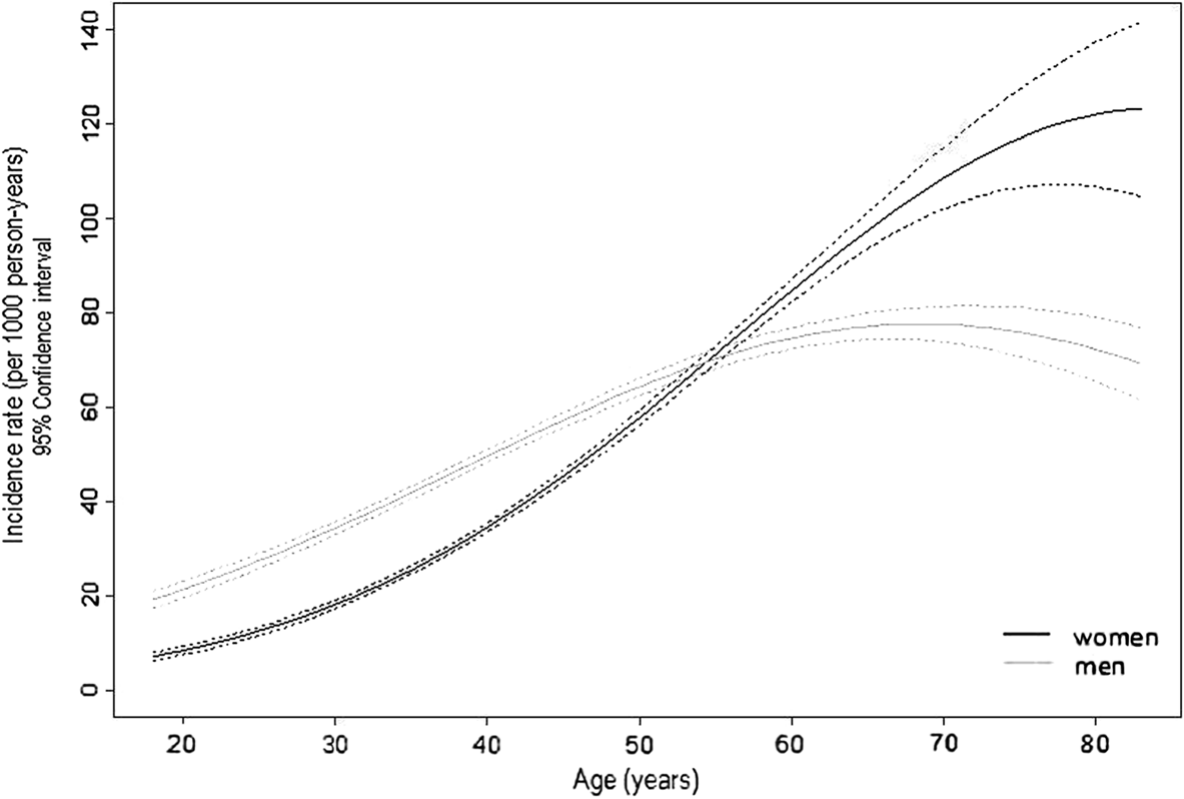
Incidence rate of hypertension according to age and gender.

During the follow-up period, 185 individuals developed hypertension, corresponding to an overall incidence rate of 47.3 (40.5-55.5) per 1000 person-years. Among women, the incidence rate was 43.4 (35.6-53.1) per 1000 person-years and among men 52.7 (41.3-68.0) per 1000 person-years. Although the incidence rate increased with age similarly in both sexes up to 60 years, with the rate being lower in women, in older participants the incidence rate stabilized among men while it continued to rise in women (Figure 2). In participants aged below 40 years, the incidence rate was 23.1 (14.2-39.9) per 1000 person-years and 40.0 (23.3-73.9), in women and men, respectively; in those aged 40 to 60 years, the rate was 53.1 (42.3-67.4) and 62.0 (47.4-82.2), and above age 60 years 110.0 (74.7-165.5) and 64.4 (39.0-110.0), among women and men, respectively. An interaction term between age and sex was statistically significant (p=0.032).

The incidence rate was similar according to marital status and decreased with increasing educational level (≥13 years vs. ≤4 years: RR=0.70, 95% CI, 0.46-1.08, p for linear trend <0.001), independently of age and sex. Overweight and obesity were associated with a 1.67-fold and 2.44-fold increased risk of hypertension, respectively, independently of age, sex and education. Current smokers and subjects with alcohol intake within the recommended range had a lower incidence rate of hypertension, but the difference was not statistically significant after adjustment for age, sex and educational level (Table 2).

**Table 2:** Incidence of hypertension by baseline characteristics of participants

	**Number of new cases**	**Incidence rate (95% CI) per 1000 person-years**	**Crude RR (95% CI)**	**Adjusted RR**^**b**^**(95% CI)**
**Education** (years)				
≤4	70	78.2 (61.8-99.6)	1	1
4–12	62	44.7 (34.0-59.8)	0.57 (0.40-0.82)	0.76 (0.52-1.01)
≥13	53	35.2 (26.5-47.8)	0.45 (0.31-0.66)	0.70 (0.46-1.08)
**Marital status**				
Married/civil union	135	52.4 (43.6-63.2)	1	1
All others	50	37.5 (28.0-51.2)	0.72 (0.51-1.02)	0.83 (0.56-1.22)
**Tobacco consumption**				
Current smoker	43	38.0 (27.6-53.7)	1	1
Ex-smoker	38	51.8 (37.1-73.6)	1.36 (0.85-2.18)	1.10 (0.67-1.79)
Never-smokers	104	53.4 (43.7-65.8)	1.40 (0.95-2.07)	
**Ethanol** (g/day)				
women: <15.0; men: <30.0	111	42.8 (35.2-52.4)	1	1
women: ≥15.0; men: ≥30.0	64	69.6 (53.8-91.1)	1.63 (1.17-2.26)	1.23 (0.86-1.76)
**Physical activity**^a^				
1^st^ third	56	43.5 (33.1-58.1)	1	1
2^nd^ third	69	56.4 (43.8-73.6)	1.30 (0.89-1.90)	1.18 (0.80-1.72)
3^rd^ third	59	44.8 (34.0-60.1)	1.03 (0.69-1.53)	1.05 (0.70-1.57)
**Body mass index** (Kg/m^2^)				
<25	64	31.2 (24.0-41.1)	1	1
25–29.9	87	64.0 (51.3-80.6)	2.05 (1.45-2.91)	1.67 (1.17-2.39)
≥30	33	94.1 (65.2-138.9)	3.02 (1.91-4.76)	2.44 (1.50-3.98)

95% CI: 95% confidence interval.

RR: relative risk.

^a^ The tertiles of the sample distribution of physical activity were 1.41, 1.58 and 3.46 MET*hour/day among women and 1.38, 1.61 and 3.26 MET*hour/day among men;

^**b**^ Adjusted for sex, age, education and an interaction term of age with sex.

## Discussion

In this urban population the incidence rate of hypertension was almost 50 per 1000 person-years, with new cases occurring predominantly among older subjects, the less educated and those with overweight-obesity. Women had lower risk than men at younger ages and higher risk after age 60.

This study provides an estimate of the incidence of hypertension in an adult Western European cohort, and it is the first longitudinal description of the adult population dynamics of the condition in Portugal. Such information will be essential to evaluate the impact of preventive measures and the potential role of changes in major risk factors, as well as to target public health objectives. However, we need to acknowledge certain limitations. Foremost, we had to rely on a relatively small sample size, because a large proportion of the baseline participants were hypertensive, confirming the large burden of high blood pressure in the Portuguese population, and because 27% of those eligible were lost to follow-up. The latter were significantly younger, less likely to be married or in a civil union, more likely to currently smoke and less likely to have high normal blood pressure when compared with the participants who were followed up. This suggests that the global incidence of hypertension reported is an overestimate of the true incidence in the population because the incidence was higher in older participants, in non-smokers and in those with high normal blood pressure who are overrepresented in our sample. Since older subjects are more likely to be non-smokers and have high normal blood pressure than the younger, with the weighting of the incidence rate according to the known age structure of the population of Porto, we corrected, at least partially, this overestimation. Additionally, the estimated proportion of adult women in the population of Porto in 2001 was lower than the proportion of women in our sample (56% in Porto [19] versus 62% in our sample), and this may have underestimated the overall incidence rate. However, weighting the analyses for sex is expected to have contributed to correct a potential selection bias in our study. Secondly, we measured blood pressure on a single occasion, both at the baseline and follow-up evaluations. Multiple measurements on different days would be a better approach to describe the true blood pressure level. Our methodology might lead to some degree of misclassification resulting in an overestimate of prevalent hypertension at baseline and incident hypertension at follow up, though this is the usual approach in epidemiological studies.

One of the first studies reporting the incidence of hypertension in the general population was the Framingham Heart Study, in 1988. Hypertension incidence per biennium ranged from 3.3% at ages 30–39 to 6.2% at ages 70–79 in men, and from 1.5% at ages 30–39 to 8.6% at ages 70–79 in women [20]. More recently, in the ARIC study, the incidence of hypertension among whites aged 45 to 64 years at baseline was 37/1000 person-years in women and 40/ 1000 person-years in men [21]. In our sample, the incidence was 63.9/1000 person-years in women and 66.5/1000 person-years in men in the same age range, around 70% higher than in the ARIC study. In Canada, using administrative data and a validated case-definition algorithm for hypertension, the age- and sex-adjusted incidence of hypertension was 32.1/1000 person-years in 2004, in participants older than 20 years [22]. In Porto Alegre, Brazil, the age- and sex-specific incidence of objectively defined hypertension in the 1990s was remarkably similar to the estimates we are reporting [23], supporting the external validity of estimates for populations sharing similar characteristics. A Spanish cohort of university graduates aged 25–65 years described a self-reported incidence rate of 8.2/1000 person-years among women, 21.8/1000 person-years among men [24] and an overall cumulative incidence of 5.8% during 4.4 years of follow-up, from 1999 to 2002 [25], which are certainly underestimates, because they relied on self-reported data. Another study performed in Spain reported estimates based on objectively measured hypertension, in participants aged at least 65 years [26]. In our sample the incidence at this age was approximately 20% higher than in the Spanish cohort, with a similar pattern of higher risk among women. Hypertension incidence rates from different studies are difficult to compare, because the estimates depend on the criteria for its definition, as well as age, gender and ethnicity composition of the study population, and the length of follow-up. However, in comparison with the estimates presented above, and despite all the described caveats, the incidence of hypertension in Portugal is very high, which is consistent with the high prevalence and high mean blood pressure previously reported in this population, contributing to maintain a high burden of disease at least in the near future [7,27].

In our sample the risk of developing hypertension varied with age, education and BMI at baseline. It is well established that the risk of hypertension depends on modifiable and non-modifiable factors. The increase in blood pressure, and consequently in the incidence of hypertension, with age has long been established, and is largely explained by the primary aging change which occurs in all societies, represented by stiffening and dilation of the proximal aorta [28]. Hypertension is arbitrarily defined by blood pressure thresholds which are reached by the vast majority of the very elderly population [18] and works just as a biomarker of disease that is more accurately described by underlying structural changes.

Like in the Framingham Heart Study [20], we observed that after the 6th decade the incidence in women becomes higher than in men. This pattern could be related with selective removal of men from the population at risk due to prevalent hypertension at baseline and lower survival due to cardiovascular deaths. It is also plausible that menopause could explain this increased risk, since it is associated with a reduction in estradiol and a decrease in estrogen-testosterone ratio, leading to endothelial dysfunction, which is common in postmenopausal women. Another putative mechanism involves sympathetic activation and increase in vasoconstriction that will cause hypertension [29]. We could not test to which extent menopause explains the sex-age pattern because this would warrant a relatively large number of both pre- and post-menopausal women within an age range compatible with menopause onset.

The higher incidence rate of hypertension in subjects with less than 4 years of schooling is consistent with those reported in the CARDIA study and the NHANES I Study [30,31] and reflects a clustering of risk factors more prevalent among the less favored people. The transition to political democracy occurring in the seventies in Portugal led to an improvement in economic capital across all social strata, but this increment was proportionally smaller among the lower socioeconomic position groups, resulting in an increasingly unequal society in the economic dimension [32]. Although there is a clear negative association between age and educational level in the Portuguese population belonging to the birth cohorts represented in this sample, the effect of education on hypertension incidence remained strong, despite marginally significant, after adjustment for age.

Blood pressure increases with weight gain, and the increasing prevalence of overweight likely contributes to the high blood pressure levels in all age groups. Fat mass could lead to hypertension by different mechanisms, namely by activating the renin–angiotensin–aldosterone system, increasing sympathetic activity, promoting insulin resistance and leptin resistance and increasing procoagulatory activity and endothelial dysfunction [33]. Our findings strengthen the substantial evidence linking obesity and overweight to the risk of hypertension [34-36].

The specific point estimates of the relative risks suggest that the associations with education, ethanol intake and BMI were stronger at younger ages (data not shown). However, the sample size did not allow us to perform an analysis stratified by age, since there were few new cases before 40 years of age, making all estimates quite unstable. This should be explored in future studies with adequate statistical power for this specific question.

## Conclusions

The annual incidence of hypertension in this adult Portuguese population was high and despite recent progresses in blood pressure related outcomes, the risk of hypertension remains higher in Portugal than in other developed countries. Changing behaviors is an urgent necessity for this population, critical to decrease the incidence of hypertension. Timely and accurate risk factor surveillance could enhance prevention and be used to monitor its effects.

## Abbreviations

95% CI: 95% confidence interval; BMI: Body mass index; MET: Metabolic equivalents; RR: Relative risk.

## Competing interests

The authors declare that they have no competing interests.

## Authors’ contributions

MP collaborated in the acquisition, analysis and interpretation of the data, and wrote the first draft of the article. NL collaborated in the design of the study and revision of the article. CP collaborated in the design of the study and revision of the article. MS collaborated in the analysis of the data. AA designed the study, analysed and interpreted the data, and reviewed the article critically for important intellectual content. HB designed the study, analysed and interpreted the data, and reviewed the article critically for important intellectual content. All authors have read and approved the final manuscript.

## Pre-publication history

The pre-publication history for this paper can be accessed here:

http://www.biomedcentral.com/1471-2261/12/114/prepub
